# Relation of Anxiety Levels and Objective Structured Clinical Examination among Undergraduate Nursing Students

**DOI:** 10.12688/f1000research.173432.3

**Published:** 2026-06-15

**Authors:** Ayman Muhammad Kamel Senosy, Gehan Sallam, Hadya Abboud Abdel Fattah, Sabah Saad El Sharkawi, Zeinab Adham Ahmed, Sara Maki, Imran Zafar, Shamsa Al Awar, Mohi Eldin Magzoub

**Affiliations:** 1 Faculty of Nursing, Ain Shams University Faculty of Nursing, Cairo, Cairo Governorate, Egypt; 2 Department of Obstetrics and Gynecology, United Arab Emirates University College of Medicine and Health Sciences, Al Ain, Abu Dhabi, United Arab Emirates; 3 Department of Nursing, Fatima College of Health Sciences, Ajman, Ajman, United Arab Emirates; 4 Faculty of Nursing, October 6 University, 6th of October City, Giza Governorate, Egypt; 5 Department of Medical Education, United Arab Emirates University College of Medicine and Health Sciences, Al Ain, Abu Dhabi, United Arab Emirates

**Keywords:** Anxiety, undergraduate, nursing students, OSCE

## Abstract

**Background:**

The incidence of stress and anxiety among nursing students is observed across all academic years of their educational training. Although Objective Structured Clinical Examinations (OSCEs) are helpful in healthcare education, they can be anxiety-inducing. This study aimed to assess the anxiety level among nursing students during the OSCE exams.

**Methods:**

A descriptive cross-sectional study was carried out on a purposive sample by distributing an online survey among students from an undergraduate nursing program. Anxiety levels were measured using the State-trait anxiety inventory, including the subscales of the state and trait of anxiety.

**Results:**

A total of 121 students with a mean age of 22.2 years (SD +/-3.6) participated in the study. The majority of the participants, 72.7%, were females, and 27.3% were males. Before attending the OSCE, 58.7% of the participants reported a mild degree of anxiety, 33.1% had a moderate level of anxiety, and only 8.3% had a severe level of trait anxiety.

**Conclusions:**

The findings highlight the complex interplay between sociodemographic factors, academic performance, work-study balance, and anxiety levels among nursing students preparing for high-stakes assessments like the OSCE. Addressing anxiety levels among nursing students preparing for OSCE assessments requires a multifaceted approach that considers individual characteristics, academic performance, and external stressors.

## Introduction

An individual’s psychological and physiological state is impacted by anxiety brought on by various physical, emotional, and cognitive factors. ^1^ Numerous researchers have studied anxiety and the mental health of undergraduate nursing students to determine their impact on the students’ psychological and academic outcomes. ^2^ Higher levels of anxiety and other mental health issues, such as stress, and depression, were reported by nursing students compared to non-nursing undergraduate students. ^3^ Stress and anxiety have been reported among healthcare professionals due to multiple sources of intrinsic stress. For example, when a person faces an excessively stressful situation, stress and anxiety can generate detrimental and long-lasting psychological effects. ^4^ Thus, measures need to be taken to prevent stress and anxiety from affecting the learning of nursing students and later the performance of nurses.

Compared to academic training, practical nurse training is reported to be much more stressful. Research has shown that an anxiety-coping program could help nursing students feel less anxious and perform better on their practical training and examination, i.e. Objective Structured Clinical Examinations (OSCEs). ^5^ This suggests that anxiety-coping programs may be used to enhance clinical activity evaluation across a range of health professions, including nursing. Therefore, educators should implement anxiety-coping programs that will aid students in lowering anxiety and enhancing their exam performance. ^5^

### Review of literature

The stress levels among nursing students during clinical training have been shown to be comparable to those observed across all academic years combined. ^6^ Although research in this regard is highly important and needed to shed light on the current situation in Middle East, the existing evidence is scarce. In the current study, stress will be defined as the degree to which a stressful event in one’s life, as well as the person’s ability to successfully deal with personal and environmental obstacles, is experienced by the individual. ^7^

The most challenging experience for many students is a relative lack of knowledge and skills. ^4^ Moreover, stressors such as fear of making mistakes, managing emergency situations, inconsistencies in clinical practice, and participation in specialized units constitute integral aspects of the initial clinical exposure. ^8^ A recent study identified a significant prevalence of exam-related anxiety among undergraduate nursing students in Jordan, with one-third experiencing high levels of anxiety and half reporting moderate anxiety levels as measured by Anxiety Scale created by. ^9^ Notably, both male and female nursing students acknowledged exam anxiety, with a third of male students experiencing severe anxiety and less than one thirds of female students reporting anxious feelings. ^10^

In addition to the demands of coursework, navigating a novel clinical setting, interacting with a diverse patient population, collaborating with nursing staff and faculty, nursing students often encounter challenges that contribute to stress during their clinical training. ^11^ Concerns about the practical application of clinical skills to deliver high-quality nursing care, apprehensions about failure, and the emotional complexities associated with patient interactions further exacerbate student anxiety. Nonetheless, several studies have explored test anxiety and coping mechanisms among students to evaluate the efficacy of employing various strategies immediately before examinations to enhance academic performance. ^12^

Nursing students are required to possess a comprehensive understanding of various subjects, encompassing multidisciplinary cooperation, client illnesses, diagnostic procedures, patient-nurse relationships, treatment modalities, and pharmacology. ^13^ The demanding academic curriculum, excessive study hours, assignments, grading pressure, time constraints, examinations, and limited social support collectively elevate stress levels among students. ^14^ Anxiety is a natural response influenced by diverse factors, as evidenced by the manifestation of test anxiety prior to exams. Students experiencing no anxiety, mild anxiety, or severe anxiety may exhibit varying impacts on their exam performance. Educators are advised to explore the factors associated with exam-related anxiety among their students based on research findings. Furthermore, implementing mind-body therapeutic interventions for students can equip them with self-management strategies to cope with high levels of anxiety effectively. ^15^

Recent OSCE-focused literature suggests that anxiety should be understood not only as an individual emotional response but also as a function of assessment design, familiarity with the format, and the adequacy of preparation. In first-year nursing students, OSCE-related stress has been associated with limited time per station, observation by examiners, waiting time, and unfamiliarity with the examination process, indicating that structural features of the OSCE itself may heighten stress levels. ^16^ Recent nursing literature also shows that although students generally perceive the OSCE as a valid and useful method for assessing clinical competence, they simultaneously describe it as stressful and demanding, particularly when preparation and orientation are insufficient. ^17^ In addition, interventional and qualitative studies suggest that structured preparation, stress-management strategies, and targeted coping programs may help reduce OSCE-related stress and improve students’ performance and psychological readiness. ^18^^,^^19^ Together, these findings indicate that OSCE anxiety is context-dependent and potentially modifiable, which supports the need for setting-specific evidence from undergraduate nursing students in the Middle East.

The researchers identified a gap in the literature regarding stress and anxiety experienced by nursing students during OSCEs, which prompted the current study. Previous evidence suggests that students who combine work and study may experience higher anxiety levels than those who do not work while studying. Similarly, students living away from their families may be more vulnerable to anxiety than those living with family support. Therefore, this study aimed to assess the anxiety levels of nursing students during the OSCE.

## Methods

The present study was carried out at the Faculty of Nursing, Six of October University, Cairo, Egypt, during the summer course period from August 1, 2022, to the end of September 2022. The research aimed to evaluate the level of anxiety preceding the OSCE exam among second-year nursing students. A descriptive study was carried out on a purposive sample, by distributing an online survey among students in an undergraduate nursing program. This group was selected because the participants were the eligible students who were about to undertake the OSCE during the study period, making them the most relevant population for examining anxiety immediately before this assessment. Anxiety levels were measured using the State-trait anxiety inventory, including the subscales of the state and trait of anxiety. A total of 180 students were approached, of whom 121 participated, yielding a response rate of 67.2%; 88 participants were female and 33 were male. The inclusion criteria were all the students in year 2 entering the OSCE exam and agreed to participate in the study. The exclusion criteria the students who refused to participate in the study or were not in year 2. After the ethical approval obtained for the study, the researchers provided detailed information to the participants, explained the study’s objectives, and distributed the questionnaire three days before the OSCE exam using an online survey method via students’ emails and WhatsApp. On average, participants took 10-15 minutes to complete the questionnaire.

### Study designs

This study employed a descriptive cross-sectional design using a purposive sample through a self-evaluation questionnaire to achieve the study objectives.

### Measures and tools

The data collection tools comprised two parts. The first part gathered socio-demographic information, encompassing six items: namely, age, gender, academic year, university entrance score, type of accommodation, and employment status.

The second part involved the State-Trait Anxiety Inventory (STAI), developed by STAI was developed by Spielberger, (1983) in English. ^16^ The questionnaire included two distinct scales, the State Anxiety (STA-Y scale) and the Trait Anxiety (TAI-Y scale). The STA-Y scale, with 20 items, assessed the participants’ current feelings at the time of responding, using a 4-point Likert scale graded as 1 (not at all), 2 (somewhat), 3 (moderately so), and 4 (very much so). Higher scores indicated high anxiety levels. The TAI-Y scale, also comprising 20 items, evaluated participants’ general feelings on a 4-point Likert scale ranging from 1 (Almost Never) to 4 (Almost Always) with item 1 being the reversed score. The total score for each subscale, i.e. the STA-Y and the TAI-Y, ranged from 20 to 80. For descriptive purposes, anxiety severity was categorized into mild, moderate, and severe levels according to previously published cut points used in comparable studies; these categories were used to facilitate interpretation and should not be considered diagnostic thresholds6.

The reliability and validity of the questionnaire were determined using Cronbach’s Alpha in this study. The reliability values of Cronbach’s Alpha were calculated for the subscales.

### Data collection

The data were collected by distributing an online survey (Annex 1) using Google Forms to students’ email addresses. Prior to the OSCE, the nursing students provided their consent for data collection. Participants were given 10-15 minutes to complete the questionnaire. No data were missing, as data collection was concluded upon reaching the predetermined sample size (n=121) within one month.

### Statistical analysis

The data were analyzed with IBM (SPSS Statistics software, version 22.0), employing both descriptive and inferential statistics. Descriptive statistics included mean, standard deviation, frequency, and percentage. Demographic and academic variables, including age, gender, university entrance score, repeating the academic year, accommodation type, and work-study status, were analyzed in relation to anxiety levels using chi-square testing. The Chi-square test was utilized to compare anxiety levels among participants before the OSCE exam with statistical significance set at P < 0.05.

The normality of the data was assessed using the Shapiro-Wilk test to determine the appropriateness of parametric testing for normally distributed data. Descriptive analysis was conducted to characterize the study sample and anxiety levels. The Chi-square test was used to identify factors associated with anxiety levels, and a two-sided P value of <0.05 was considered statistically significant.

Continuous normally distributed data were presented in mean ± standard deviation (SD), while categorical data were expressed as frequencies and percentages. The Chi-square test (Fisher’s exact test when applicable) was used for comparing variables with categorical data. The reliability of the questionnaires used in the study was assessed through internal consistency, with Cronbach’s alpha values of 0.894 for the STA-Y scale and 0.886 for the TAI-Y scale indicating good reliability. The questionnaire was administered in English.

### Ethical considerations

This study received ethical approval from the Faculty of Nursing on October 6 University’s ethical committee, with approval number: PRC-Nu-2308002.

### Informed consent

The study’s objectives were clearly explained and simplified for the participants. Confidentiality regarding any shared information was assured. Participants were informed of their right to withdraw from the study at any time. Written informed consent was obtained from the participants before the study commenced.

## Results

Table 1 showed the mean age of the participants, 22.2 ±3.6, distributed as 78.5% ranging from 19 to 25 years and 21.5% aged ≤ 18 years. Gender distribution showed that 72.7% of the participants were females and 27.3% were males. Regarding the university entrance average score, 56 participants (46.3%) scored ≤ 75%, 52 (43.0%) scored between 76% – 89%, and 13 (10.7%) scored ≥ 90%. Academic progress indicated that 62 participants (51.2%) repeated the academic year, while 59 (48.8%) did not. Concerning the type of accommodation, 62.0% were living in private housing, 7.4% in university dormitories, and 30.6% were living with family. Additionally, work-study balance showed that 40 participants (33.1%) worked and studied, while 81 (66.9%) did not engage in both activities simultaneously.

**Table 1.** Demographic characteristics of the participating students (n = 121).

	N	%
**Age (Years)**		
18	26	21.5
19 – 25	95	78.5
**Mean ± SD**	22.2 ± 3.6	
**Gender**		
Female	88	72.7
Male	33	27.3
**University entrance average score**		
≤75%	56	46.3
76%–89%	52	43
≥90%	13	10.7
**Do you repeat this academic year (Level)?**		
Yes	62	51.2
No	59	48.8
**Kind of accommodation**		
Private	75	62
University dormitory	9	7.4
Living with family	37	30.6
**Do you work and study?**		
Yes	40	33.1
No	81	66.9

Table 2 includes the classification of anxiety levels into mild, moderate, and severe anxiety for both the STA-Y and TAI-Y scales of anxiety. The data shows the distribution of participants across these categories: for mild anxiety, 46.3% based on the STA-Y scale and 58.7% based on the TAI-Y scale; for moderate anxiety, 40.5% according to the STA-Y scale and 33.1% according to the TAI-Y scale; and for severe anxiety, 13.2% based on the STA-Y scale and 8.3% based on the TAI-Y scale. Additionally, the mean anxiety scores were 49.4 ± 9.3 for the STA-Y classification and 48.4 ± 8.0 for the TAI-Y classification.

**Table 2.** Comparison between the studied students’ anxiety levels based on the State-trait anxiety inventory (STAI), classified as the state (STA-Y scale) and trait (TAI-Y scale) of anxiety (n = 121).

	STA-Y classification		TAI-Y classification	
	n	%	N	%
**Anxiety Level**				
Mild Anxiety	56	46.3	71	58.7
Moderate Anxiety	49	40.5	40	33.1
Severe Anxiety	16	13.2	10	8.3
**Mean ± SD**	49.4 ± 9.3		48.4 ± 8.0	

STA-Y = State of anxiety, TAI-Y = trait of anxiety.

As shown in Table 3, there was no statistically significant relation between the total level of anxiety according to STA-Y classification and sociodemographic characteristics among studied students; specifically, age, gender, repeating the academic year, and kind of accommodation (P > 0.05), while the university entrance score and combining working and studying were significantly correlated with STA-Y (P < 0.001).

**Table 3.** Relation between total level of anxiety according to STA-Y classification and demographic characteristics among studied students (n=121).

	Mild Anxiety (n=56)		Moderate Anxiety (n=49)		Severe Anxiety (n=16)			
	N	%	n	%	n	%	X ^2^	P
**Age (Years)**								
18	12	21.4	11	22.4	3	18.8		
19–25	44	78.6	38	77.6	13	81.3	0.098	0.952
**Gender**								
Female	46	82.1	31	63.3	11	68.8		
Male	10	17.9	18	36.7	5	31.3	4.842	0.088
**University entrance average score**								
≤75%	7	12.5	35	71.4	14	87.5		
76%–89%	36	64.3	14	28.6	2	12.5		
≥90%	13	23.2	0	0.0	0	0.0	53.208	<0.001 **
**Do you repeat this academic year (Level)?**								
Yes	30	53.6	22	44.9	10	62.5		
No	26	46.4	27	55.1	6	37.5	1.723	0.423
**Kind of accommodation**								
Private	34	60.7	30	61.2	11	68.8		
University dormitory	2	3.6	7	14.3	0	0.0		
Living with family	20	35.7	12	24.5	5	31.3	6.622	0.157
**Do you work and study?**								
Yes	9	16.1	19	38.8	12	75.0		
No	47	83.9	30	61.2	4	25.0	20.744	<0.001 **

** P value is highly significant, <0.001.

Similar patterns were found in the relation between sociodemographic factors and TAI-Y ( Table 4).

**Table 4.** Relation between total level of anxiety regarding TAI-Y classification and demographic characteristics among studied students (n=121).

	Mild Anxiety (n = 71)		Moderate Anxiety (n = 40)		Severe Anxiety (n = 10)			
	N	%	n	%	n	%	X ^2^	P
**Age (Years)**								
18	16	22.5	8	20.0	2	20.0		
19–25	55	77.5	32	80.0	8	80.0	0.112	0.946
**Gender**								
Female	56	78.9	26	65.0	6	60.0		
Male	15	21.1	14	35.0	4	40.0	3.373	0.185
**University entrance average score**								
≤75%	18	25.4	28	70.0	10	10.0		
76%–89%	40	56.3	12	30.0	0	0.0		
≥90%	13	18.3	0	0.0	0	0.0	35.787	<0.001 **
**Do you repeat this academic year (Level)?**								
Yes	37	52.1	17	42.5	8	80.0		
No	34	47.9	23	57.5	2	20.0	4.555	0.103
**Kind of accommodation**								
Private	40	56.3	28	70.0	7	70.0		
University dormitory	5	7.0	4	10.0	0	0.0		
Living with family	26	36.6	8	20.0	3	30.0	4.308	0.366
**Do you work and study?**								
Yes	7	9.9	23	57.5	10	10.0		
No	64	90.1	17	42.5	0	0.0	48.315	<0.001 **

** P value is highly significant, <0.001.

## Discussion

The study aimed to assess the anxiety levels of nursing students during OSCEs. The findings indicated that the majority of students exhibited mild to moderate anxiety, as classified by both STA-Y and TRI-Y. While sociodemographic factors did not significantly correlate with anxiety levels based on STA-Y classification, university entrance scores and combining work and study were significantly associated. Similar patterns were observed for TAI-Y classification, highlighting the complex interplay between academic performance, work-study balance, and anxiety levels in nursing students preparing for OSCEs.

The sociodemographic data and characteristics of the students in this study indicate that more than three-quarters of the participants fall within the age range of 19 to 25 year with a majority being females. This observation aligns with previous studies highlighting the relationship between students’ age. ^17^ Regarding repeating a year of study, the current research revealed that nearly half of the students are at the same study level for the second time as they failed in their previous trial for the same OSCE exam, as a result of the other factors as being working or living away from their families. This could be attributed to the challenges students face in their studies and exams, impacting their ability to progress successfully. ^17, 18^ In terms of accommodation while studying at university, the study showed that nearly two-thirds of the students reside in private housing which emphasizes the importance of suitable living arrangements for student well-being and academic performance. Furthermore, concerning employment during their studies, this study indicates that approximately two-thirds of the students do not work alongside their studies. Balancing work and study commitments can significantly influence students’ stress levels and academic performance, particularly before high-stakes assessments like the OSCE. This association may reflect the competing demands of balancing academic responsibilities with employment; however, because socioeconomic status was not directly measured in this study, no conclusions can be drawn regarding its role in the observed anxiety levels. ^19^

In comparing the anxiety levels of the students based on STA-Y and TAI classifications, this study reveals that nearly half of the students exhibit mild anxiety, with approximately one-third experiencing moderate anxiety, and less than one-quarter facing severe anxiety. The mean score of both STA-Y and TAI-Y classifications were 49.4 ± 9.3 and 48.4 ± 8, respectively. These findings are supported by recent scientific studies, ^19, 20^ indicating a substantial proportion of students experiencing mild to moderate state anxiety before the OSCE. This prevalence may be associated with students lacking coping mechanisms and stress-relief strategies, ^21, 22^ particularly as they approach the OSCE for the first time. As OSCEs contribute to high levels of stress and anxiety among students due to various factors associated with these assessments, ^23^ research underscores the importance of addressing anxiety management strategies among nursing students to enhance their performance and well-being during high-pressure assessments like the OSCE.

In terms of the relation between the total level of anxiety according to STA-Y and TAI-Y classifications and demographic characteristics among the studied students, the study’s findings indicate there is a highly significant correlation with university entrance score and the combination of working and studying (P≤0.001). This finding is consistent with Ref. 24 cognitive factors contributing to students’ test anxiety during OSCE assessments, and Ref. 25 which highlighted a moderate and significant direct correlation between self-efficacy scores and OSCE performance. Students reported that confidence plays a crucial role in reducing anxiety levels and improving self-perceived performance. These findings may indicate that students who combine work and study experience additional academic and personal demands. ^26^ However, because socioeconomic status was not measured directly, the present study cannot determine whether socioeconomic factors contributed to the observed associations. ^27^

### Implications and recommendations

The presented study highlights some recommendations to improve the anxiety level among undergraduate students.

• To implement coping anxiety program on the nursing students before OSCE examination.
• To provide an orientation session to the nursing students before the OSCE exam to reduce their anxiety level.
• To identify the more anxious students during the mock OSCE tests and support them through motivational interview before the OSCE.
• To identify the predictive anxiety factors during mock OSCE tests.

### Limitations

The main limitation of this study was the inclusion of students solely from a single university and medical discipline (nursing), thereby restricting the external validity and generalizability of our findings. Furthermore, the anxiety coping program was not executed, and no follow-up assessment on student anxiety levels were carried out. Future research endeavors should encompass diverse medical disciplines from different universities, extend the duration of the interventions, and incorporate more follow-up assessments to enhance the robustness and applicability of the study outcomes. Additionally, the cross-sectional design precludes causal inference. Furthermore, prior exposure to OSCEs, number of previous attempts, and degree of preparation were not directly measured and may have acted as confounding factors affecting anxiety levels. Another limitation is that anxiety was assessed using a self-report questionnaire, which may be subject to bias or social desirability bias. In addition, anxiety levels were measured at a single time point, three days before the OSCE, and therefore may not fully reflect students’ anxiety immediately before or during the examination. Because participation was voluntary and data were collected through an online survey, response bias cannot be excluded, and participants may have differed from non-participants in their anxiety levels.

## Conclusions

The findings illustrate the relationship between sociodemographic characteristics, academic achievement, work-study balance, and anxiety levels among nursing students preparing for high-stakes assessments such as the Objective Structured Clinical Examination (OSCE). Managing anxiety among nursing students preparing for OSCE examinations necessitates a multimodal strategy that takes into account individual characteristics, academic achievement, and external stresses. Healthcare institutions, policymakers, educators, and researchers may all help to improve student well-being, academic achievement, and overall performance in high-pressure assessment settings by introducing focused techniques and support systems.

Healthcare institutions and educators are recommended to prioritize implementing anxiety management strategies tailored to nursing students facing OSCE assessments. Providing support mechanisms and coping strategies can enhance students’ performance and well-being during stressful evaluation periods. In addition, developing policies that support students balancing work and study commitments can help alleviate stress and improve academic outcomes. Moreover, universities and nursing schools might need to consider integrating wellness programs into the curriculum to address anxiety among students. Incorporating stress-relief techniques and mental health support can enhance student resilience and performance during challenging assessments. Future research should focus on longitudinal studies to explore the long-term effects of anxiety on nursing students’ academic progression and professional practice. Understanding the persistent impact of anxiety can guide interventions and support mechanisms for students.

This article is available as a preprint in Research Square. ^28^

## Data Availability

Figshare. Relation of Anxiety Levels and Objective Structured Clinical Examination among Undergraduate Nursing Students. https://doi.org/10.6084/m9.figshare.30693638.v2. ^29^ This project contains the following underlying data:
•
**Data.xlsx** (De-identified numerical data for all variables included in the analysis, including the values used to generate all tables and summary statistics). •
**STA.docx** (State Anxiety Inventory questionnaire used for data collection). •
**TAI.docx** (Trait Anxiety Inventory questionnaire used for data collection). Data are available under the terms of the CC0 license.
