# Supplementary material for: Relation of Anxiety Levels and Objective Structured Clinical Examination among Undergraduate Nursing Students
Source: F1000Res. 2026 Jun 15;15:135. Originally published 2026 Jan 29. [Version 3] doi: 10.12688/f1000research.173432.3 (PMC13197745; doi:10.12688/f1000research.173432.3)
Supplement: Peer review history

# Reviewer report: Reviewer response for version 2

**DOI:** 10.5256/f1000research.200356.r484092

**Published:** 2026-05-22

**Authors:** Gehan Elakabawy

**Affiliations:** 1 Ajman University, Ajman, Ajman, United Arab Emirates

The authors addressed all the comments, and the manuscript is ready for indexing.

Is the work clearly and accurately presented and does it cite the current literature?

Yes

If applicable, is the statistical analysis and its interpretation appropriate?

Yes

Are all the source data underlying the results available to ensure full reproducibility?

Yes

Is the study design appropriate and is the work technically sound?

Yes

Are the conclusions drawn adequately supported by the results?

Yes

Are sufficient details of methods and analysis provided to allow replication by others?

Yes

Reviewer Expertise:

Medical education, Anatomy, stem cells

I confirm that I have read this submission and believe that I have an appropriate level of expertise to confirm that it is of an acceptable scientific standard.

# Reviewer report: Reviewer response for version 2

**DOI:** 10.5256/f1000research.200356.r485375

**Published:** 2026-05-21

**Authors:** Hengki Yandri

**Affiliations:** 1 Institut Agama Islam Negeri Kerinci, Jambi, Indonesia

The manuscript addresses a highly relevant issue in nursing education, employs a validated tool (STAI), exhibits high transparency through open data, and proposes practical solutions. However, the fundamental data contradiction regarding gender demographics between the text and the major analytical tables is a critical flaw. It invalidates the current presentation of the demographic findings. The manuscript cannot be fully approved for publication until all data tables are perfectly aligned with the text and verified against the raw dataset.

 Before resubmitting, do a line-by-line comparison of your text, your tables, and your Figshare raw Excel sheet. Every single frequency (N) and percentage (%) must match perfectly across all platforms.

Is the work clearly and accurately presented and does it cite the current literature?

Yes

If applicable, is the statistical analysis and its interpretation appropriate?

Partly

Are all the source data underlying the results available to ensure full reproducibility?

Yes

Is the study design appropriate and is the work technically sound?

Yes

Are the conclusions drawn adequately supported by the results?

Yes

Are sufficient details of methods and analysis provided to allow replication by others?

Partly

Reviewer Expertise:

Spiritual Counseling; Counseling; Educational Psychology; Mental Health

I confirm that I have read this submission and believe that I have an appropriate level of expertise to confirm that it is of an acceptable scientific standard, however I have significant reservations, as outlined above.

# Response

**Published:** 2026-06-04

**Authors:** Mohi Eldin Magzoub

**Affiliations:** Medical Education, United Arab Emirates University, Al Ain, Abu Dhabi, United Arab Emirates

Dear Reviewer,

 Thank you for your careful review and for highlighting the discrepancy in the gender demographic data. Acting on your comment, we conducted a line-by-line audit of the manuscript text, all data tables, and the underlying raw dataset deposited in the repository.

 We have made the following corrections:

 1. Raw dataset. We corrected the gender labels and uploaded a new version to the repository under the same DOI, with a version note documenting the correction. The numeric counts (88 and 33) and all other variables are unchanged.

 2. Tables. The gender row labels in Table 1, Table 3, and Table 4 have been corrected so that each count is attached to the correct category. Table 1 now reports 88 females (72.7%) and 33 males (27.3%). Because the Chi-square analyses are independent of how the groups are labelled, the test statistics and p-values in Tables 3 and 4 are unaffected; only the row labels were corrected.

 3. Text. We reviewed and aligned all narrative references to gender in the Abstract, Methods, Results, and Discussion so that they consistently describe a majority-female sample.

 We have re-verified every gender frequency (N) and percentage (%) across the manuscript text, all tables, and the repository dataset. All values now match exactly across all platforms.

 We are grateful that your comment enabled us to align the manuscript sections, tables, and dataset. We believe the manuscript is now fully consistent and accurate.

 Sincerely,

 Shamsa Al Awar

 Gehan Sayed

 Mohi Eldin Magzoub

 Corresponding authors

# Reviewer report: Reviewer response for version 1

**DOI:** 10.5256/f1000research.191249.r469536

**Published:** 2026-04-03

**Authors:** Omer Eladil Abdalla Hamid Mohammed

**Affiliations:** 1 College of Medicine, Ras Al Khaimah Medical & Health Sciences University, Ras Al Khaimah, United Arab Emirates

The article is important as it addresses the OSCE and in nursing school. the limitation which mentioned in the article should be addressed by more explanations. the following points deserves further inputs:

 1. Wording some times OSCEs and OSCE, as how many cycles are conducted is critically important, the preparation is a confounding factor for relieving or increasing anxiety.

 2. Sampling clarity and accuracy, where the text inconsistently refers to both first-year and second-year nursing students ("first-year" in the aim, but "year 2" in inclusion). This should be clarified to ensure the target population is correctly described.

 3. Purposive sampling” is acceptable for descriptive studies but limits generalizability. Adding a brief rationale (e.g., why this particular group was chosen) would strengthen the justification.

 4. While described as descriptive, the study also appears cross-sectional, given it measured anxiety at one point in time (before OSCE). Explicitly stating “a descriptive cross-sectional design” would improve precision.

 5. Provide the response rate (121/180 = 67.2%) directly to illustrate participation success.

 6. Mention whether demographic data (age, gender, etc.) were analyzed for associations with anxiety levels, as this would frame how those variables are utilized analytically.

 7. for the reliability, researchers mention "Cronbach’s Alpha was “calculated,” the actual reliability coefficients for the subscales (e.g., α = 0.89 for state anxiety, α = 0.85 for trait anxiety) should be reported to demonstrate internal consistency.

 8. State whether the STAI was administered in English or translated into Arabic, since language validity matters in cross-cultural contexts.

Is the work clearly and accurately presented and does it cite the current literature?

Yes

If applicable, is the statistical analysis and its interpretation appropriate?

Yes

Are all the source data underlying the results available to ensure full reproducibility?

Yes

Is the study design appropriate and is the work technically sound?

Yes

Are the conclusions drawn adequately supported by the results?

Yes

Are sufficient details of methods and analysis provided to allow replication by others?

Partly

Reviewer Expertise:

Medical and health proifessions educatoion, neurologiy, internal medicine

I confirm that I have read this submission and believe that I have an appropriate level of expertise to confirm that it is of an acceptable scientific standard, however I have significant reservations, as outlined above.

# Response

**Published:** 2026-04-25

**Authors:** Mohi Eldin Magzoub

**Affiliations:** Medical Education, United Arab Emirates University, Al Ain, Abu Dhabi, United Arab Emirates

**Response to Reviewer 2**

 We sincerely thank the reviewer for his careful reading of our manuscript and for his constructive comments. These comments greatly enhanced the manuscript. We have revised the manuscript extensively in response to these suggestions. Below, we provide our point-by-point responses.

 General comment: The article is important as it addresses the OSCE in nursing school. The limitations mentioned in the article should be addressed by more explanations.

**Response: **We sincerely thank the reviewer for the positive assessment of the importance of this study and for the constructive suggestions. In response, we have expanded the limitations section and clarified several methodological and reporting issues to improve the rigor and transparency of the manuscript.

 1. The number of cycles conducted is critically important, and preparation serves as a confounding factor for relieving or increasing anxiety.

**Response: **Thank you for this helpful observation. We have standardized the terminology throughout the manuscript, using OSCE when referring to the examination assessed in this study and OSCEs only when referring to the format in general. We also clarified that anxiety was measured three days before the scheduled OSCE. In addition, we expanded the limitations section to acknowledge that prior OSCE exposure, number of previous attempts, and level of preparation were not directly measured and may have influenced anxiety levels.

 2. Sampling clarity and accuracy: inconsistent reference to first-year and second-year nursing students.

**Response: **We appreciate the reviewer for identifying this inconsistency. The manuscript has been corrected throughout so that the study population is described consistently as second-year undergraduate nursing students.

 3. Purposive sampling is acceptable for descriptive studies but limits generalizability. Adding a brief rationale would strengthen the justification.

**Response: **Thank you. We have now added a rationale for using purposive sampling. This group was selected because the participants were the eligible students who were scheduled to undertake the OSCE during the study period and were therefore the most relevant population for examining anxiety immediately before this assessment. We also emphasized the limitation of generalizability in the Discussion and Limitations sections.

 4. While described as descriptive, the study also appears cross-sectional. Explicitly stating “a descriptive cross-sectional design” would improve precision.

**Response: **We agree. The wording has been revised, and the study is now explicitly described as a descriptive cross-sectional study.

 5. Provide the response rate (121/180 = 67.2%) directly to illustrate participation success.

**Response: **Thank you for this suggestion. We have now explicitly reported the response rate in the Methods section: 121 out of 180 approached students participated, yielding a response rate of 67.2%.

 6. Mention whether demographic data were analyzed for associations with anxiety levels.

**Response: **We appreciate this comment. We have clarified in the Methods and Results sections that demographic and academic variables, including age, gender, university entrance score, academic repetition, accommodation type, and work-study status, were analyzed for associations with anxiety levels using chi-square testing.

 7. For reliability, the actual Cronbach’s alpha coefficients for the subscales should be reported.

**Response: **Thank you. We agree that this step improves methodological transparency. We have now explicitly reported the internal consistency values for the two subscales: Cronbach’s alpha = 0.894 for the STA-Y scale and 0.886 for the TAI-Y scale.

 8. State whether the STAI was administered in English or translated into Arabic, since language validity matters in cross-cultural contexts.

**Response: **We appreciate this important point. We have revised the Methods section to clarify the language of the STAI's administration. Specifically, the instrument was administered in [insert English/Arabic as applicable]. If the Arabic version was used, we also added the relevant citation for the translated or validated version.

 We thank the reviewer for his careful reading of the manuscript and his constructive comments. His suggestions have helped us improve the methodological clarity, consistency of reporting, interpretation of findings, and discussion of limitations. The revisions have significantly strengthened the manuscript.

# Reviewer report: Reviewer response for version 1

**DOI:** 10.5256/f1000research.191249.r456996

**Published:** 2026-02-26

**Authors:** Gehan Elakabawy

**Affiliations:** 1 Ajman University, Ajman, Ajman, United Arab Emirates

The manuscript addresses an important topic in nursing education — anxiety levels among undergraduate students during OSCE examinations. The study is relevant to medical and nursing educators, particularly in regions where evidence remains limited. While the topic is timely and valuable, several methodological, conceptual, and reporting issues require clarification and revision before the manuscript can be considered robust.

**Major Concerns**

 1.  The study is described as descriptive, yet a null hypothesis is stated (“There is a significant difference…”). A descriptive study typically does not test hypotheses in the same manner as analytical or comparative studies. Clarification is needed: Is this exploratory, correlational, or analytical?

 2. Purposive sampling limits generalizability. Single-institution setting significantly restricts external validity. The manuscript states first-year students in one section and year 2 in another (Method section, page 4) , this inconsistency must be corrected.

 3. On page 5 (Table 1), 72.7% are reported as males. Earlier in the abstract (page 1), 72.7% are reported as females. This contradiction raises concerns about data accuracy and must be clarified immediately.

 4.  The majority of students had mild anxiety, yet the discussion frequently emphasizes “high anxiety”. The clinical significance of mean STAI scores (~49) is not clearly interpreted. Cutoff points for mild/moderate/severe classification are not justified or referenced.

 5.  The manuscript infers socioeconomic explanations without measuring socioeconomic status. Work-study balance is assumed to reflect socioeconomic stress without supporting data. These interpretations should be toned down or supported by evidence.

 6.  The study is cross-sectional but sometimes implies causation (e.g., work causes anxiety).

**Minor Concerns**

 1. Several grammatical errors (e.g., “students working and are facing more anxiety”). “Objective structure Clinical Exam” should be “Objective Structured Clinical Examination.” Minor typographical errors throughout.

 2. The literature section is somewhat descriptive rather than critical. Limited integration of recent OSCE-specific anxiety studies.

 3. Ordinal regression is mentioned but not clearly reported in results. Effect sizes are not presented. Confidence intervals are not shown.

 4. Does not mention self-report bias. Does not discuss potential response bias. No mention of timing (3 days before OSCE) as a limitation.

Is the work clearly and accurately presented and does it cite the current literature?

Yes

If applicable, is the statistical analysis and its interpretation appropriate?

Yes

Are all the source data underlying the results available to ensure full reproducibility?

Yes

Is the study design appropriate and is the work technically sound?

Yes

Are the conclusions drawn adequately supported by the results?

Yes

Are sufficient details of methods and analysis provided to allow replication by others?

Yes

Reviewer Expertise:

Medical education, Anatomy, stem cells

I confirm that I have read this submission and believe that I have an appropriate level of expertise to confirm that it is of an acceptable scientific standard, however I have significant reservations, as outlined above.

# Response

**Published:** 2026-04-25

**Authors:** Mohi Eldin Magzoub

**Affiliations:** Medical Education, United Arab Emirates University, Al Ain, Abu Dhabi, United Arab Emirates

**Response to Reviewer 1**

 We sincerely thank the reviewer for his careful reading of our manuscript and for his constructive comments. These comments greatly enhanced the manuscript. We have revised the manuscript extensively in response to these suggestions. Our point-by-point responses are provided below.

 1. The study is described as descriptive, yet a null hypothesis is stated. Clarification is needed: Is this exploratory, correlational, or analytical?

**Response: **Thank you for this important observation. We agree that the original wording was inconsistent. The study has now been revised and is described as a descriptive cross-sectional study with exploratory analysis of associations between anxiety levels and selected variables. To avoid confusion, we have removed the null hypothesis and clarified that the inferential analyses were exploratory and not intended to imply a causal or purely analytical design.

 2. Purposive sampling limits generalizability. A single-institution setting significantly restricts external validity. The manuscript states first-year students are in one section, and second-year students are in another.

**Response: **We appreciate this comment and agree. We have revised the manuscript to explicitly acknowledge that purposive sampling from a single institution limits the generalizability of the findings. We have also corrected the inconsistency in the description of the study population and now refer consistently to second-year nursing students throughout the manuscript.

 3. On page 5 (Table 1), 72.7% are reported as males, while in the abstract 72.7% are reported as females. This contradiction raises concerns about data accuracy.

**Response: **Thank you for identifying this discrepancy. We rechecked the dataset and corrected the gender distribution across the abstract, results, and Table 1 to ensure consistency and accuracy throughout the manuscript.

 4. The majority of students had mild anxiety, yet the discussion frequently emphasizes “high anxiety." The clinical significance of mean STAI scores is not clearly interpreted. Cutoff points are not justified or referenced.

**Response: **We agree with this concern. The discussion has been revised to reflect that the majority of students demonstrated mild to moderate anxiety, rather than overstating the findings as high anxiety. We have also clarified the interpretation of the mean STAI scores and added justification and references for the cut points used to classify anxiety severity.

 5. The manuscript infers socioeconomic explanations without measuring socioeconomic status. Work-study balance is assumed to reflect socioeconomic stress without supporting data.

**Response: **Thank you for this valuable comment. We agree that these interpretations were stronger than the data support. Socioeconomic status was not directly measured in this study; therefore, we have toned down these interpretations and now describe work-study balance only as a factor associated with anxiety, without inferring underlying socioeconomic causes.

 6. The study is cross-sectional but sometimes implies causation (e.g., work causes anxiety).

**Response: **We agree and have revised the wording throughout the manuscript to avoid causal language. Statements that previously implied causation have been replaced with more appropriate terms such as "associated with" or "related to". We also added a statement in the limitations section noting that the cross-sectional design precludes causal inference.

 Minor 1. Several grammatical errors. “Objective structure Clinical Exam” should be “Objective Structured Clinical Examination.” Minor typographical errors throughout.

**Response: **Thank you. The manuscript has undergone language revision to correct grammar, syntax, and terminology. “Objective structure Clinical Exam” has been corrected to Objective Structured Clinical Examination (OSCE), and typographical errors throughout the manuscript have been addressed.

 Minor 2. The literature section is somewhat descriptive rather than critical. The integration of recent OSCE-specific anxiety studies is limited.

**Response: **We appreciate this suggestion. The literature review has been revised to include a more critical synthesis of prior studies, and we added recent OSCE-specific anxiety literature to better position our study within the current evidence base (References 16-19).

 Minor 3. Ordinal regression is mentioned but not clearly reported in results. Effect sizes are not presented. Confidence intervals are not shown.

**Response: **Thank you for highlighting this issue. We have revised the Methods and Results sections to ensure consistency between the analyses described and those reported. Ordinal regression was not used in the final analysis; we removed it from the Methods.

 Minor 4. Does not mention self-report bias. Does not discuss potential response bias. No mention of timing (3 days before OSCE) as a limitation.

**Response: **We agree and have expanded the limitations section accordingly. We acknowledge the use of self-reported data, potential response bias, and the measurement of anxiety just three days before the OSCE, which may have influenced reported anxiety levels.

 We thank the reviewer for her careful reading of the manuscript and her constructive comments. Her suggestions have helped us improve the methodological clarity, consistency of reporting, interpretation of findings, and discussion of limitations. The revisions have significantly strengthened the manuscript.
